# The Roles of Apoptosis in Swine Response to Viral Infection and Pathogenesis of Swine Enteropathogenic Coronaviruses

**DOI:** 10.3389/fvets.2020.572425

**Published:** 2020-11-26

**Authors:** Zhichao Xu, Yun Zhang, Yongchang Cao

**Affiliations:** ^1^State Key Laboratory of Biocontrol, School of Life Science, Sun Yat-sen University, Guangzhou, China; ^2^Higher Education Mega Center, School of Life Science, Sun Yat-sen University, Guangzhou, China

**Keywords:** swine enteropathogenic coronaviruses, transmissible gastroenteritis virus, *porcine epidemic diarrhea virus*, *porcine deltacoronavirus*, swine acute diarrhea syndrome coronavirus, swine, apoptosis

## Abstract

Apoptosis is a tightly regulated mechanism of cell death that plays important roles in various biological processes including biological evolution, multiple system development, anticancer, and viral infections. Swine enteropathogenic coronaviruses invade and damage villous epithelial cells of the small intestine causing severe diarrhea with high mortality rate in suckling piglets. *Transmissible gastroenteritis virus* (TGEV), *Porcine epidemic diarrhea virus* (PEDV), *Porcine deltacoronavirus* (PDCoV), and *Swine acute diarrhea syndrome coronavirus* (SADS-CoV) are on the top list of commonly-seen swine coronaviruses with a feature of diarrhea, resulting in significant economic losses to the swine industry worldwide. Apoptosis has been shown to be involved in the pathogenesis process of animal virus infectious diseases. Understanding the roles of apoptosis in host responses against swine enteropathogenic coronaviruses infection contribute to disease prevention and control. Here we summarize the recent findings that focus on the apoptosis during swine coronaviruses infection, in particular, TGEV, PEDV, PDCoV, and SADS-CoV.

## Introduction

Apoptosis, also known as programmed cell death, is a ubiquitous mode of cell death known to be responsible for clearance of unwanted, injured, or virus-infected cells (1, 2). Cells undergoing apoptosis are accompanied by characteristic morphological changes, including cell shrinkage and deformation, chromatin condensation, nuclear fragmentation, and plasma membrane blebbing, which forms the apoptotic body containing the fragments of nucleus or cytoplasm (3). Cell apoptosis is an active process, which involves a series of genes activation and expression, various proteins regulation. To date, it was reported that there are two main apoptotic pathways: the extrinsic/death receptor pathway (4) and the intrinsic/mitochondrial pathway (5). Death receptors belong to the tumor necrosis factor receptor (TNFR) superfamily (6). Members of the TNFR family are type I membrane surface receptors, which include the Fatty acid synthetase receptor (FasR), TNFR, Death receptor (DR)3, DR4, DR5, etc. (7–11). Their ligands belong to type II membrane proteins, which include Fatty acid synthetase ligand (FasL), TNF-α Apo3 ligand (Apo3L), Apo2 ligand (Apo2L), etc. (7–11). Upon death receptor-ligand binding, the adapter protein Fas-associated death domain (FADD) is recruited by death domain, then associates with pro-cysteinyl aspartic acid protease (caspase)-8 *via* dimerization of the death effector domain and a death-inducing signaling complex (DISC) is formed to activate caspase-8, then caspase-8 results in the caspase-3 activation (12–14). Once caspase-3 is activated, the execution phase of apoptosis is triggered (12–14). The mitochondrial pathways involve some chemical or physical stimulus factors leading to change of the permeability of mitochondrial membrane, resulting in release of the cytochrome *c* (cyt *c*) or other apoptotic molecules into the cytoplasm cavity and activation of downstream caspases to initiate apoptosis (15, 16). In addition, T-cell mediated cytotoxicity involved with perforin-granzyme to kill target cells is known as another apoptotic pathway (17).

It is known that many viruses can evolve various sophisticated strategies to modulate apoptosis as a critical armament to complete their replication cycle (18), reflected in the relationship between viral infection and cell apoptosis is bidirectional. Viruses could hijack host's apoptotic pathway to delay apoptotic response, providing sufficient time for maximizing progeny virus production (19). On the other hand, viruses could induce apoptosis to enable the release and dissemination of viral progeny for further invasion to the neighboring cells at the late stages of viral infection (18).

**Figure 1:**
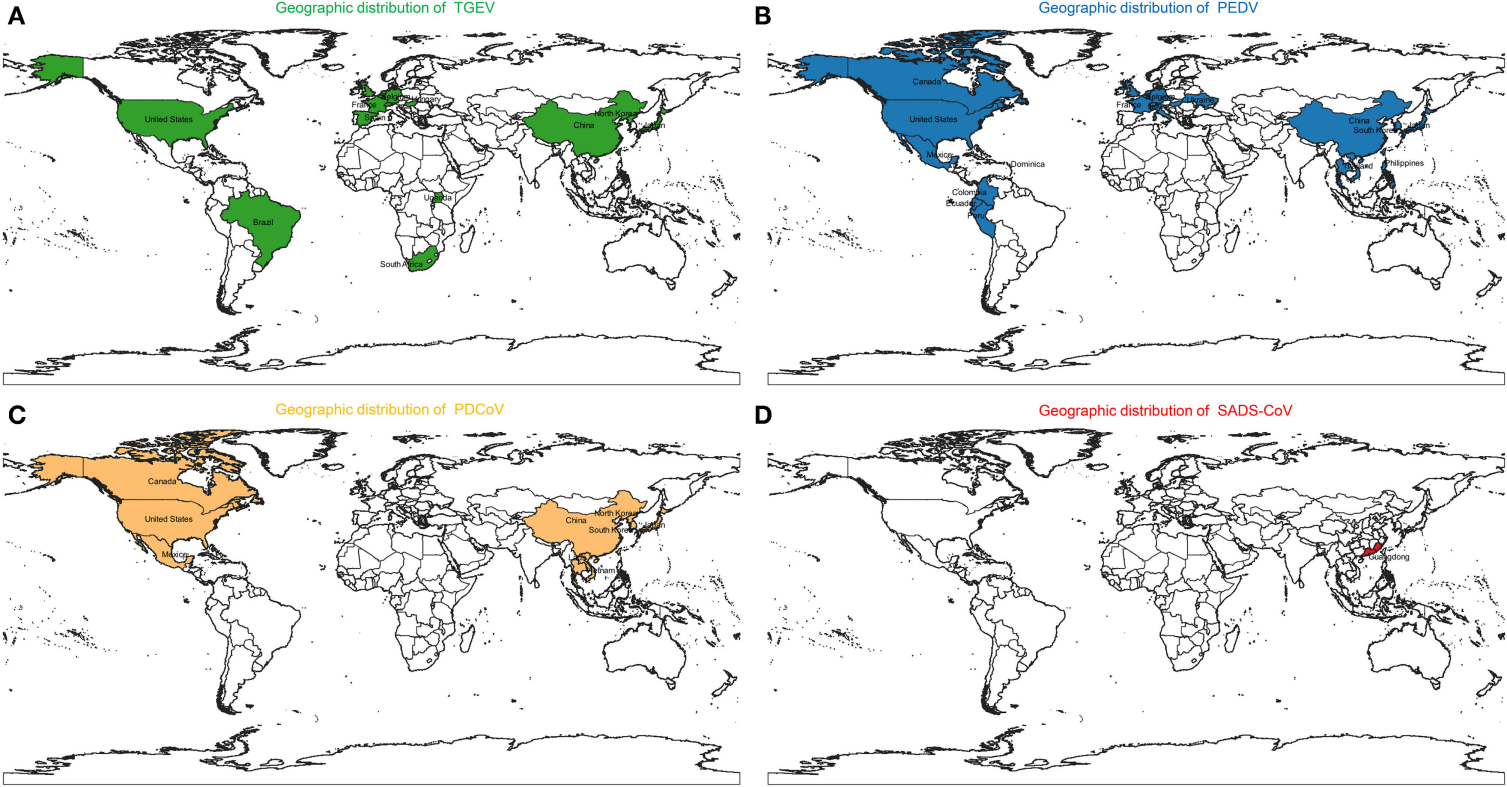
Geographic distribution of **(A)** *Transmissible gastroenteritis virus* (TGEV), **(B)** *Porcine epidemic diarrhea virus* (PEDV), **(C)** *Porcine deltacoronavirus* (PDCoV), and **(D)** *Swine acute diarrhea syndrome coronavirus* (SADS-CoV).

Porcine coronaviruses (CoVs) are significant enteric and respiratory pathogens of swine. Six porcine CoVs have so far been identified: *transmissible gastroenteritis virus* (TGEV) (20), *porcine respiratory coronavirus* (PRCV) (21), *porcine epidemic diarrhea virus* (PEDV) (22), and *swine acute diarrhea syndrome coronavirus* (SADS-CoV) (23) in the *Alphacoronavirus* genus; porcine hemagglutinating encephalomyelitis virus (PHEV) (24) in the *Betacoronavirus* genus; *porcine deltacoronavirus* (PDCoV) (25) in the *Deltacoronavirus* genus. The clinical signs of swine enteropathogenic CoVs including TGEV, PEDV, PDCoV, and SADS-CoV are characterized by severe watery diarrhea with subsequent dehydration in pigs of all ages, and a high mortality rate in suckling piglets (26–29). The molecular surveillance studies indicated that swine enteropathogenic CoVs were common viral pathogen of pigs around the world (30–44) (Figure 1). In addition, co-infections of these diarrhea-associated viruses were commonly found in pig farms (45). For swine enteropathogenic CoVs infection prevention, the administration of vaccines and antiviral drugs are important tool. Currently a number of vaccines and antiviral drugs, such as killed, live-attenuated vaccine, shRNA expression vector are widely used to prevent swine enteropathogenic CoVs infection (46–49). However, it still can not stop the swine enteropathogenic CoVs outbreak, due to they are not optimal in terms of safety and efficacy, and large-scale infections still occur, resulting in the death of large numbers of piglets, causing huge economic losses to the pig industry (23, 50–52), indicating that a deeper understanding of the pathogenesis of swine enteropathogenic CoVs is needed to develop more effective prevention and control measures.

Although swine enteropathogenic CoVs might infect multiple organs in pigs, the intestinal tract is the major target organ, where virus replication is limited to intestinal villus epitheial cells (29, 53). Diarrhea caused by pathogen infection associates with viral damage to intestinal epithelial cells, which plays an important role in the nutrition absorption (54), causes a breach of mucosal physical barriers and reduction of enzyme activities, leading to electrolyte imbalances, nutrient decomposition and absorption anomalies (53, 55). It has been reported that apoptosis, which occurs in the infection course of many CoVs (56, 57), is involved in viral pathogenesis and disease processes that promote cell death and tissue injury (58, 59). In this review, the roles of apoptosis in the pathogenesis and control of TGEV, PEDV, PDCoV, and SADS-CoV will be discussed, which may provide some clues to further understandings of pathogenesis of swine enteropathogenic CoVs.

## Apoptosis Associated With *Transmissible Gastroenteritis Virus* (TGEV)

### Virus Characteristics of TGEV

TGEV is an enveloped, single-stranded, positive-sense RNA virus with a genome of appropriately 28 kb in length (20, 60). The full-length genome of TGEV is arranged in the order of: 5′ UTR-ORF1a/1b-S-3a-3b-E-M-N-7-3′ UTR, containing nine open reading frames (ORFs) encoding four structure proteins (S, M, N, E) and five non-structure proteins (61). The nsp1 protein of TGEV can efficiently suppress protein synthesis in mammalian (62). The nsp3 protein of TGEV can cleave a peptide mimicking the cognate nsp2|nsp3 cleavage site based on its papain-like protease 1 (PL1(pro)) domain (63). The N protein of TGEV belongs to a multifunctional phosphoprotein, which can package the RNA genome into a helical ribonucleoprotein, regulate viral RNA synthesis, and modulate of host cell metabolism (64). E protein promotes TGEV maturation in the secretory pathway (65). S1 and M proteins play a role in viral replication (66, 67). In addition, M protein can affect TGEV-induced IFN-α production (68).

### The Role of Apoptosis in TGEV Infection

**Figure 2:**
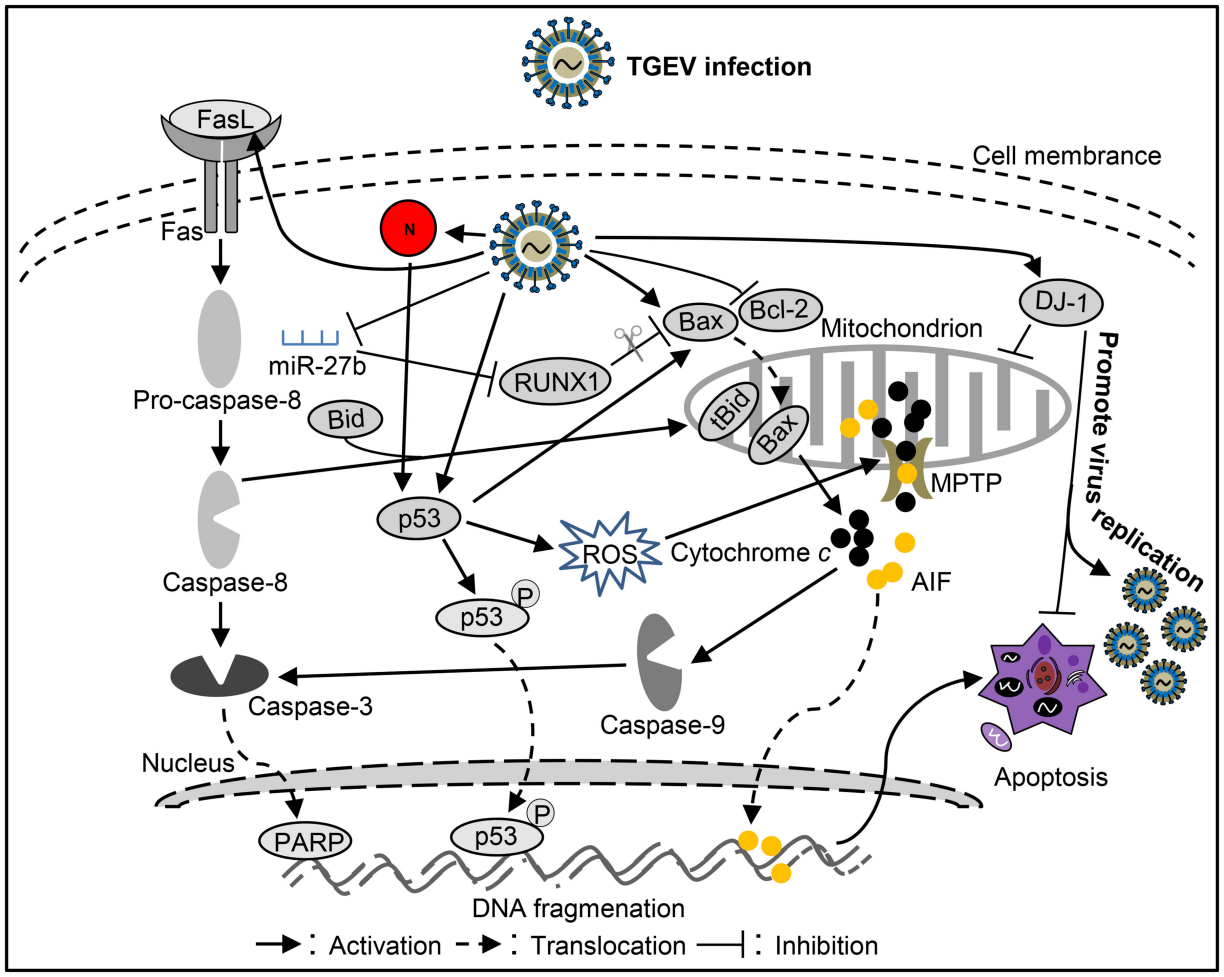
Diagram of the roles of apoptosis in the pathogenesis of *Transmissible gastroenteritis virus* (TGEV) infection.

It was reported that TGEV invades and replicates in villous epithelial cells to provoke villous atrophy, causing severe diarrhea, and dehydration in piglets is the central event in the pathogenesis of TGEV infection (69, 70). Apoptosis plays a important role in the pathogenesis process of animal virus infectious diseases (71–73). Many studies shown that TGEV infection could induce apoptosis in PK-15 cells, swine testicular (ST) cells, swine kidney cells, MDCK-APN cells (canine kidney cell line expressing porcine APN) or human rectal tumor cells (HRT18, expressing porcine APN) (Table 1) (74–82, 91), which associates with intracellular molecules, such as p53, reactive oxygen species (ROS), mitochondrial apoptosis-inducing factor (AIF), poly (ADP-ribose) polymerase (PARP), and caspases (74, 82). Interestingly, TGEV may not induce apoptotic death of intestinal villous enterocytes *in vivo* (77). TGEV infection could decrease p300/CBP, down-regulate MDM2, and promote p53 phosphorylation at serine 15, 20, and 46, resulting in accumulation and activation of p53, then p53 induced ROS accumulation which leads to mitochondrial oxidative damage to release cyt *c* to the cytosol and thereby activate apoptosis in PK-15 cells (79, 91). In addition, TGEV infection up-regulated FasL to activate caspase-8 and cleaved Bid to tBid which was transferred to the mitochondria, resulting in release of cyt *c* into the cytoplasm to activate caspase-9 (78). Moreover, TGEV could down-regulate Bcl-2, increase the expression of Bax, and promote the transfer of Bax from cytoplasm to mitochondria (78). Then, mitochondria released cyt *c* to activate caspase-9, and finally caspase-9 activates caspase-3 to induce cell apoptosis (78). microRNAs (miRNAs) play a key role in the regulation of virus-induced apoptosis (80). For instance, miR-27b can directly target the 3′ UTR of runt-related transcription factor 1 (RUNX1) mRNA to regulate the expression of RUNX1 in PK-15 cells (80). It has been found that TGEV infection can down-regulate the expression of miR-27b in host cells, thereby down-regulating the expression of RUNX1 and activating the Bax regulated expression of caspase-9/3 to induce cell apoptosis (80). These results suggest that TGEV can induce apoptosis through both extrinsic and intrinsic pathways (Figure 2) in PK-15 cells. Further analysis of the key viral proteins induced by TGEV showed that N protein could activate p53, p21 to eliminate cyclin B/cdc2, promote the transfer of Bax to mitochondria, lead to the release of cyt *c* from mitochondria, and activate caspase-9/3 to induce cell apoptosis (81) (Figure 2). Interestingly, N protein was cleaved at the position of VVPD359 by activated caspase-6/7 during TGEV-induced apoptosis (76) in human rectal tumor cell line, indicating that N protein plays a very important role in the interaction between virus and host during apoptotic process. It is well-known that viruses could manipulate apoptosis to complete their life cycle. It was reported that mitophagy induced by DJ-1 to counteract apoptosis could promote viral infection during TGEV infection (54) (Figure 2). The above studies confirmed that there may be a certain correlation between pathogenicity and apoptosis after TGEV infection. More research is needed on how apoptosis affects the proliferation and spread of TGEV during viral infection.

**Table 1:** Compare apoptotic cell death caused by the four swine enteropathogenic CoVs and the related mechanisms.

**Virus**	**Genomic organization**	**Cell lines used in apoptotic studies**	**Virus-related apoptotic cell death occurs *in vitro* or *in vivo***	**Apoptotic pathway**	**Apoptosis involved molecules**	**Contribution of apoptosis to virus replication**	**References**
TGEV	5′ UTR-ORF1a/1b-S-3a-3b-E-M-N-7-3′ UTR	PK-15, IPEC-J2, HRT18, ST cells	*In vitro*	Extrinsic and intrinsic	miR-27b, RUNX1, Bax, Caspase 3/8/9, DJ-1, AIF, p53, ROS, FasL, Bax, PARP, p53, AIF	No effect	(54, 61, 74–82)
PEDV	5′ UTR-ORF1a/1b-ORF2-ORF3-ORF4-ORF5-ORF6-3′ UTR	IECs, Vero, Marc-145 cells	*In vitro* and *in vivo*	Extrinsic and intrinsic	Caspase 3/8, AIFM1, PARP, p53, ROS, AIF	Facilitate	(18, 53, 83–85)
PDCoV	5′ UTR-ORF1a/1b-S-E-M-ns6-N-ns7 3′ UTR	LLC-PK, ST cells	*In vitro*	Intrinsic	Bax, Caspase3/9, Cyt *c*, PARP	Facilitate	(86–89)
SADS-CoV	5′ UTR-ORF1a/1b-S-NS3-E-M-N-NS7a-3′ UTR	Vero, IPI-2I cells	*In vitro*	Extrinsic and intrinsic	Fas, Caspase3/8/9, Bax, Cyt *c*, PARP	Facilitate	(23, 90)

## Apoptosis Associated With *Porcine Epidemic Diarrhea Virus* (PEDV)

### Virus Characteristics of PEDV

PEDV is an enveloped, single-stranded, positive-sense RNA virus with a genome of appropriately 28 kb in length (22). The viral genome is sequentially composed of 5′ untranslated region (UTR), open reading frame 1a/1b (ORF1a/1b), ORF2-6, and 3′ UTR (84). The ORF 1a/1b cover the 5′-proximal two-thirds of the genome coding for replicase polyprotein (pp) la and pp1ab, respectively (84, 92). These pp1a and pp1ab polyproteins can be cleaved by internal proteases generating sixteen non-structural proteins, namely nsp1-16 (85). Moreover, the genome of PEDV encodes four structural proteins including the spike (S), envelope (E), membrane (M), and nucleocapsid (N) proteins, while ORF3 encodes an accessory protein (84). The functional form of the S protein is a trimer, which protrude from the viral membrane thus providing typical crown appearance of the CoVs (93). It functions during cell entry by binding to cellular receptors and causing fusion of the viral and host cell membranes (93). During maturation, the S protein is cleaved into a receptor-binding subunit S1 and a membrane-fusion subunit S2 (84). The E protein has ion channel activity and plays an important role in virion morphogenesis (93, 94). The M protein is the main component of the viral envelope and interacts with all structural components during viral particle assembly (93). The N protein packages the genomic RNA to form the helical nucleocapsid (RNP) (93). ORF3 protein was known to be related to PEDV pathogenicity (93). In addition, N and ORF3 are involved in viral replication (95, 96). Furthermore, the encoded N, M, E, ORF3, PLP2, nsp 1, nsp 3, nsp 5, nsp 7, nsp 14, nsp 15, nsp 16 proteins can antagonize Interferon-β production (97–100).

### The Role of Apoptosis in PEDV Infection

**Figure 3:**
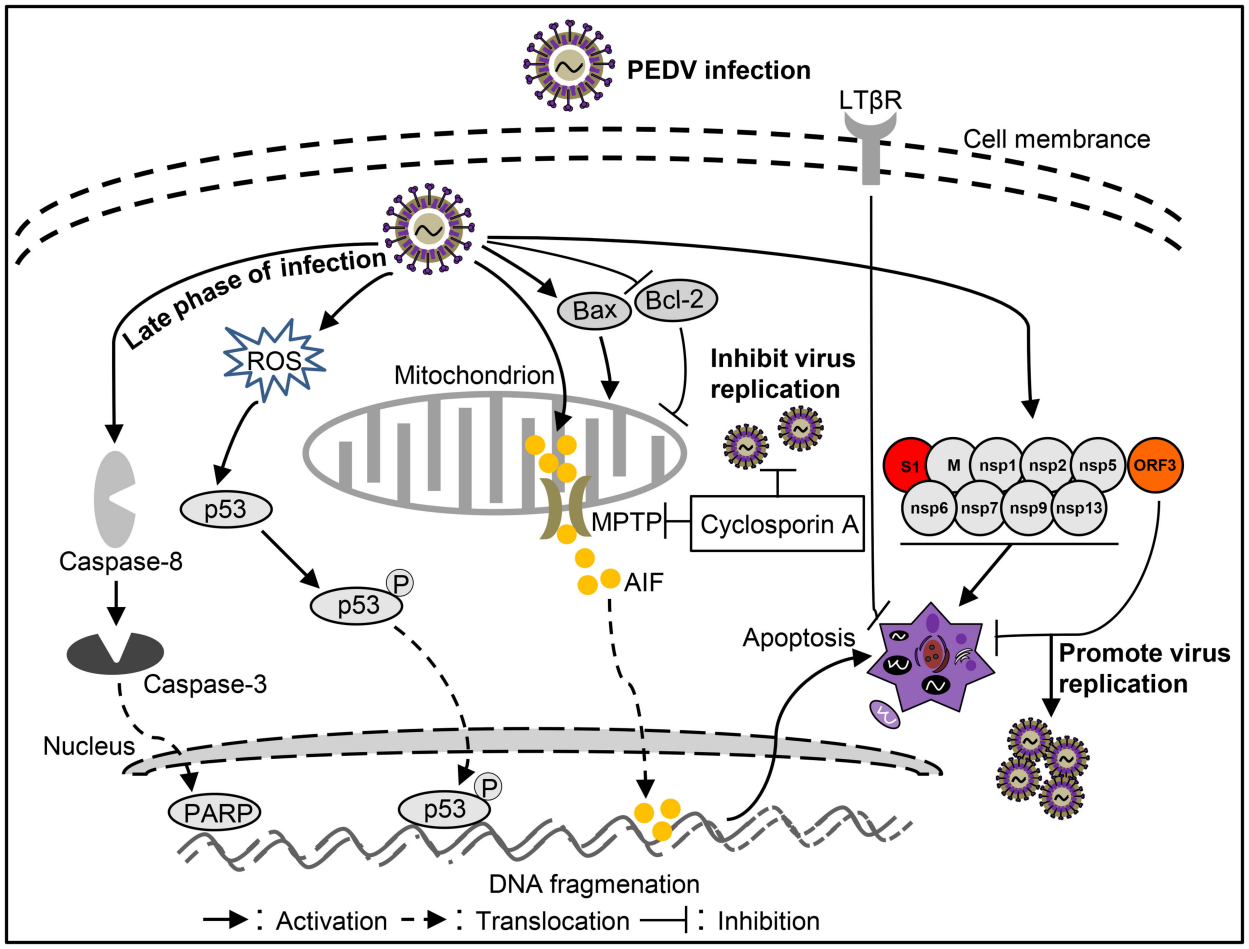
Diagram of the roles of apoptosis in the pathogenesis of *Porcine epidemic diarrhea virus* (PEDV) infection.

PEDV infection can damages pig intestinal epithelial tissue and interfere with epithelial mucosal cell function, resulting in abnormal nutrient absorption and diarrhea (53). This phenomenon may be related to apoptosis caused by PEDV infection. Apoptosis of cells in the lamina propria or submucosa of PEDV-infected jejunum or ileum was increased (18, 101). However, PEDV may not induce apoptosis death of intestinal villous enterocytes *in vivo* (50), like TGEV and PDCoV. AIF could translocate to the nucleus to cause apoptosis after PEDV infection in Vero cells (18). Moreover, PEDV could promote p53 phosphorylation at serine 20 and subsequent translocation to the nucleus, leading to p53 activation and thereby apoptosis in Vero cells (83). During this process, ROS also accumulates to promote apoptosis (83). In addition, apoptosis was mediated by activation of caspase-8 and caspase-3 in the late stage of PEDV-infected Vero cells (102). Treatment with the inhibitor of pro-apoptotic molecule could significantly inhibit PEDV infection (18), indicating that apoptosis plays an important role in the PEDV pathogenicity. However, the host cells showed an anti-apoptotic effect through LTBR during PEDV infection (103). By analysis cell apoptosis induced by PEDV-related viral proteins, it was found that M, nsp1, nsp2, nsp5, nsp6, nsp7, nsp9, nsp13, and S1 proteins can induce apoptosis, among which S1 is the critical apoptotic-inducing protein in Vero cells, but the detailed molecular mechanism is still unclear (85). On the contrary, PEDV encoded ORF3 could inhibit cell apoptosis to promote virus proliferation (93), indicating that PEDV applies different strategies to regulate cell apoptosis in different stages of infection to complete viral proliferation. Although some studies have been conducted on the influence of PEDV on apoptosis (Figure 3), many questions remain unclear, such as how does S1 induce apoptosis? And what role does S1 portein induced-apoptosis play in virus-caused diarrhea? Clarifying these issues will help explain how PEDV causes diarrhea in pigs.

## Apoptosis Associated With *Porcine Deltacoronavirus* (PDCoV)

### Virus Characteristics of PDCoV

PDCoV is an enveloped, single-stranded, positive-sense RNA virus with a genome of appropriately 25 kb in length (26). The genome organization of PDCoV are in the order of: 5′ untranslated region (UTR), open reading frame 1a/1b (ORF1a/1b), spike (S), envelope (E), membrane (M), non-structural protein 6 (ns6), nucleocapsid (N), non-structural protein 7 (ns7), and 3′ UTR (86, 87). The diverse dimerization forms of nsp9 protein could enhance their nucleic acid binding affinity (104). ns6 protein is an important virulence factor of PDCoV (105). It is known that N, nsp5, nsp15, ns6 contribute to inhibit interferon-β production (106–109).

### The Role of Apoptosis in PDCoV Infection

**Figure 4:**
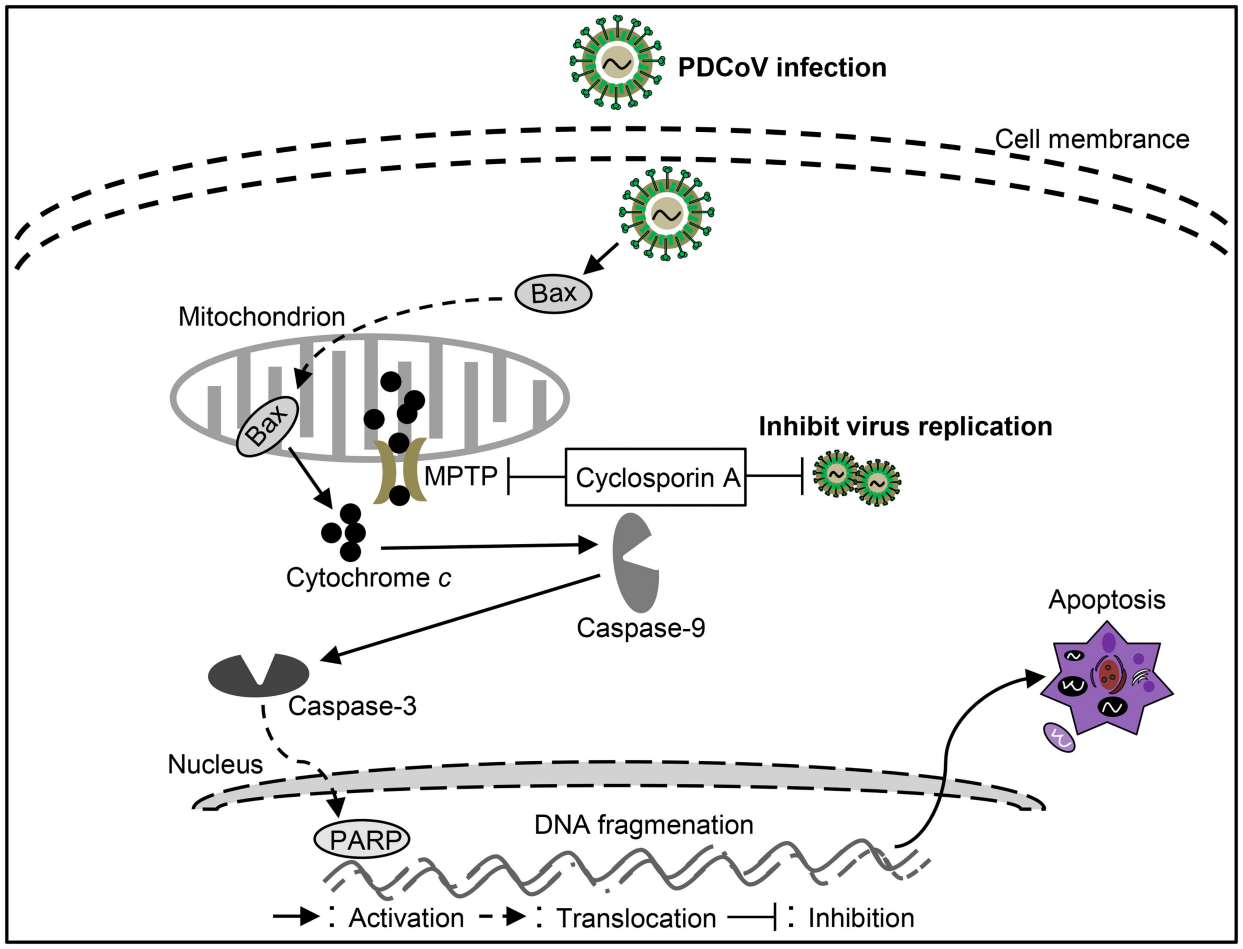
Diagram of the roles of apoptosis in the pathogenesis of *Porcine deltacoronavirus* (PDCoV) infection.

PDCoV is a newly discovered virus that causes severe clinical diarrhea and intestinal pathological damage in piglets (88), but the pathogenesis of PDCoV infection is still largely unknown. Current studies showed that PDCoV infection could promote Bax translocation and mediate mitochondrial outer membrane permeabilization (MOMP), resulting in specific relocation of the mitochondrial cyt *c* into the cytoplasm, thus activating caspase-9/3 to initiate apoptosis in ST cells (88). These results indicate that PDCoV mediates cell apoptosis through a caspase-dependent endogenous apoptotic pathway (Figure 4). Moreover, apoptosis caused by PDCoV contributes to viral protein translation and the caspase-dependent intrinsic apoptosis pathway in PDCoV-infected ST cells is also used for facilitation of viral replication (88). Interestingly, PDCoV induces apoptosis in swine testicular and LLC porcine kidney cell lines *in vitro* but not in infected intestinal enterocytes *in vivo*. Another form of cell death, necrosis, has been found in PDCoV-infected swine intestinal enterocytes as well as in the porcine small intestinal epithelial cell line, IPEC-J2 *in vitro* (89). The above results indicate that a better model of cellular infection is needed to reflect the infection *in vivo*. In-depth study on the molecular mechanism of cell death caused by PDCoV infection will help to analyze the pathogenesis of PDCoV. In addition, the key proteins responsible for cell death caused by the virus still need to be further studied, which will help to identify virulence factors and provide guidance for prevention and control PDCoV infection.

## Apoptosis Associated With *Swine Acute Diarrhea Syndrome Coronavirus* (SADS-CoV)

### Virus Characteristics of SADS-CoV

SADS-CoV, also named PEAV (110) and SeACoV (111), is an enveloped, single-stranded positive-sence RNA virus (110). The full-length genome of SADS-CoV is about 27 kb (110), arranged in the order of: 5′ UTR-ORF1a/1b-S-NS3-E-M-N-NS7a-3′ UTR (112). It is known that the S protein has many important characteristics in CoVs, such as virus attachment and entry, and induction of neutralizing antibodies *in vivo* (113). Of note, compare to other reported CoVs, SADS-CoV has the smallest S protein including 1,129 amino acids (110). To date, the papain-like protease 2 (PLP2) domain of nsp3 was shown to be able to cleave nsp1 proteins and also peptides mimicking the nsp2/nsp3 cleavage site and also have deubiquitinating and deISGynating activity (114). The function of other viral proteins of SADS-CoV remains to be further explored.

### The Role of Apoptosis in SADS-CoV Infection

**Figure 5:**
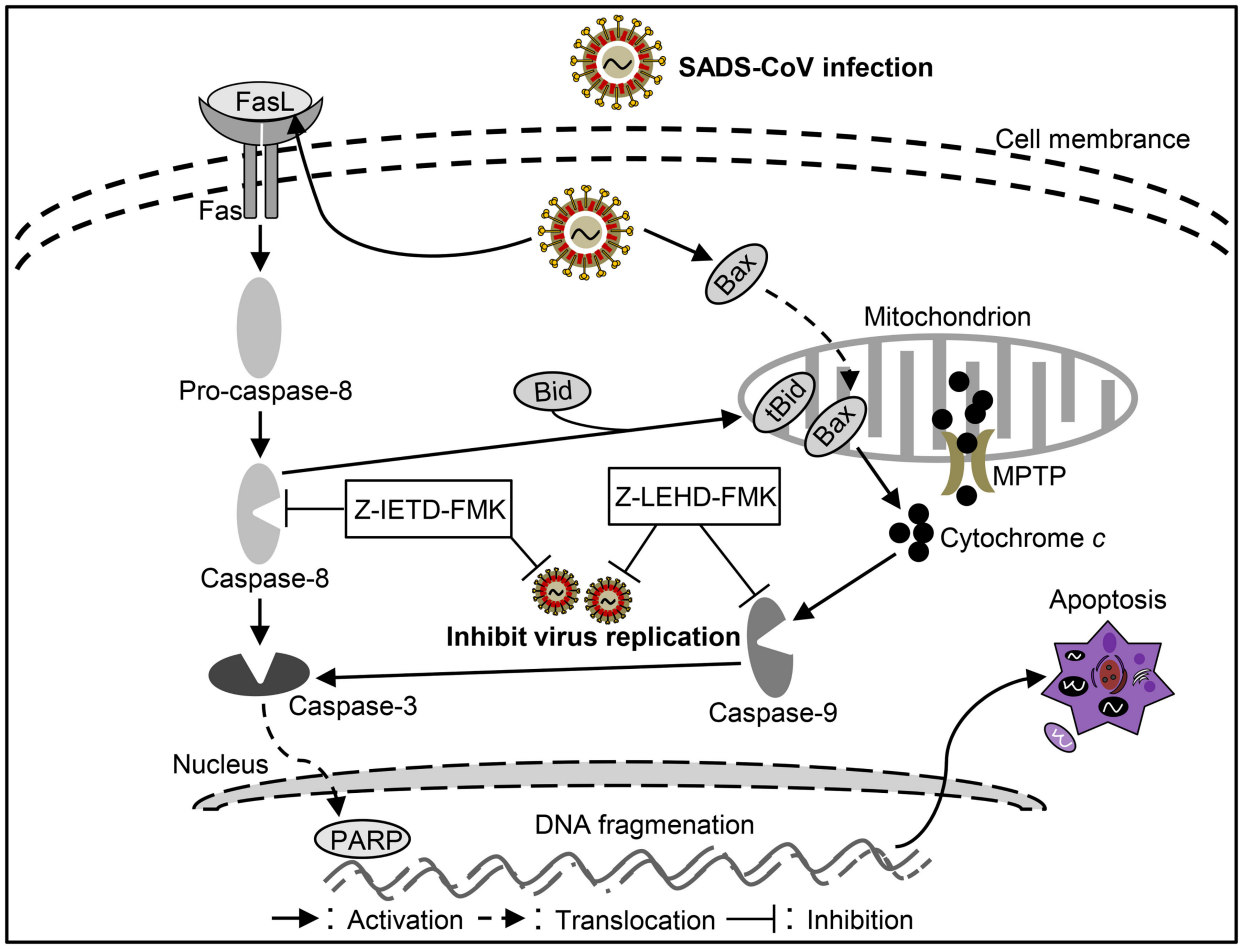
Diagram of the roles of apoptosis in the pathogenesis of *Swine acute diarrhea syndrome coronavirus* (SADS-CoV) infection.

As another newly identified swine intestine CoV, detail information of the pathogenic mechanism of SADS-CoV remains unclear. It was reported that SADS-CoV infection could increased apoptosis in the small intestinal epithelial cell line IEC *in vitro* (90). SADS-CoV infection could up-regulate FasL, subsequentially activates caspase-8/3 to lead to apoptosis in Vero and IPI-2I cells (90). Moreover, activated caspase-8 could cleave Bid, then the cleaved Bid translocated to mitochondria participating in the destruction of mitochondria integrity and cyt *c* release to cytosol, which in turn facilitates caspase-9/3 activation thus result in apoptosis (90). In addition, SADS-CoV infection triggers Bax recruitment into the mitochondria, leading to cyt *c* but not AIF release into cytoplasm to induce apoptosis through mitochondrial permeability transition pore (MPTP), which involve with cyclophilin D (CypD) in these processes (90). These results suggest SADS-CoV-induced apoptosis were mediated by both extrinsic and intrinsic pathways (Figure 5). The viral replication was affected with the inhibitors of caspase-8 or capases-9, indicating that SADS-CoV-induced apoptosis contributes to viral replication (90). Although it has been demonstrated well in SADS-CoV induced apoptosis, the function of viral protein in SADS-CoV-induced apoptosis and the exact mechanism underlying remains unclear. More efforts to elucidate the molecular mechanisms of SADS-CoV-induced apoptosis will help to explore the pathogenesis of SADS-CoV infection.

## Other Mechanisms Are Related to the Pathogenesis of Swine Enteropathogenic CoVs

Innate immunity is thought to be the first line of host defense against a wide variety of pathogenic infections (115). Of note, type I interferon (IFN-α/β), as important cytokines of innate immunity induced by virus invasion, could establish an anti-viral state in infected sites (116). In order to infect the organism and cause pathogenicity, the virus must break through the anti-viral state of the organism. It was reported that PEDV, PDCoV and SADS-CoV can inhibit the up-regulated expression of type I interferon through a variety of different mechanisms (100, 115, 117–120), thus leading to virus infection, indicating that the inhibition of type I interferon might relate to the pathogenesis of these viruses. In addition, inhibition of anti-viral status to promote viral infection might contribute to the occurrence of apoptosis (121). Interestingly, unlike PEDV, PDCoV, and SADS-CoV, TGEV infects the body can promote the up-regulated expression of IFN-β (122). IFN-β has been reported to induce apoptosis (123, 124). Whether the apoptosis induced by TGEV is related to the upregulation of IFN-β needs further study.

## Conclusions

Lines of evidence indicate that apoptosis play critical roles in the pathogenesis of swine enteropathogenic CoVs. Most of the information is gleaned from the studies on the apoptosis of TGEV, PEDV, PDCoV, or SADS-CoV infections *in vitro* and *in vivo*. Viral proteins, such as N from TGEV, S1 from PEDV, are involved in regulation of virus-mediated apoptosis production, which might provide some clues to determine the virulence factors and serve as the targets of antiviral drugs. However, the roles of apoptosis in host response to swine enteropathogenic CoVs infection alone or co-infections are still far from elucidation and need to be further investigated. In addition, the apoptotic forms and mechanisms caused by these four swine enteropathogenic CoVs are different (Table 1), which whether involved with the differences of pathogenicity also needs further study. In brief, further investigation into the role of apoptosis in these swine enteropathogenic CoVs is conducive to elucidate of the pathogenesis of viral infections and develop an appropriate strategy for the prevention and control of swine diarrhea diseases.

## Author Contributions

ZX collected the data and wrote the paper. YZ revised the paper. YC checked and finalized the manuscript. All authors read and approved the final manuscript.

## Conflict of Interest

The authors declare that the research was conducted in the absence of any commercial or financial relationships that could be construed as a potential conflict of interest.
